# Effective pupil dilatation with a mixture of 0.75% tropicamide and 2.5% phenylephrine: A randomized controlled trial

**DOI:** 10.4103/0301-4738.55070

**Published:** 2009

**Authors:** Adisak Trinavarat, Auengporn Pituksung

**Affiliations:** Department of Ophthalmology, Faculty of Medicine Siriraj Hospital, Mahidol University, Bangkok, Thailand

**Keywords:** Mydriatric, phenylephrine, pupil dilatation, tropicamide

## Abstract

**Purpose:**

To compare the efficacy in pupil dilatation between a mixture containing 0.75% tropicamide and 2.5% phenylephrine and the alternate application of 1% tropicamide and 10% phenylephrine eye drops.

**Material and Methods:**

Patients requiring pupil dilatation were randomized to receive one drop of the mixture every 10 min for four times or our standard application of one drop of 1% tropicamide alternating with one drop of 10% phenylephrine every 10 min for two cycles. Pupil size was measured under bright light with the pupil gauge before, and every 5 min after initial application for 40 min. Application of the drops was discontinued once the pupil diameter reached 7 mm. Blood pressure and pulse rate were monitored every 15 min.

**Results:**

Of 40 patients (age 57.3±10.9 years, range 35-70 years), 22 were randomized into the mixture group and 18 into the alternate drug group. Baseline pupil sizes were 1.7±0.5 mm in the mixture group and 1.8±0.4 mm in the alternate drug group. The pupils were successfully dilated to 7 mm within 40 min in 17 patients of the mixture group compared to seven patients in the alternate drug group (*P*=0.004, Log Rank test). The mean pupil sizes at 40 min were 6.6±0.8 and 6.0±0.9 mm in the mixture and alternate drug groups respectively (*P*=0.050, t-test). Blood pressure and pulse rate were stable and similar in both groups.

**Conclusions:**

The mixture of 0.75% tropicamide and 2.5% phenylephrine is superior to our standard application of 1% tropicamide alternating with 10% phenylephrine. It provides faster and more successful pupil dilatation within 40 min.

Pupil dilatation is mandatory for thorough indirect ophthalmoscopy. Drugs used to dilate the pupil include parasympatholytic and sympathomimetic agents. We routinely used 1% tropicamide and 10% phenylephrine in our patients for this purpose. The former was instilled followed by the latter 10 min later. This cycle was repeated 10 min after the first one. In case the pupil was not adequately dilated, another cycle of instillation was applied. Occasional adverse drug reactions have been reported with the usage of 10% phenylephrine, and consists of tachycardia, elevated blood pressure and stroke.[1] Sequencing of drugs in this alternate application routine was sometimes confusing in our busy clinic.

To address these problems, we prepared a mixture containing 0.75% tropicamide and 2.5% phenylephrine to use as a mydriatric agent. This study evaluated the efficacy in pupil dilatation of this mixture compared to the alternate application of the separate drugs according to our standard practice.

## Materials and Methods

This double-blind randomized controlled trial was approved by the institutional ethical committee. This study was conducted at the outpatient unit, Department of Ophthalmology, Faculty of Medicine Siriraj Hospital, Mahidol University, Thailand. Patients requiring pupil dilatation for indirect ophthalmoscopy were enrolled. Exclusion criteria included being younger than 20 years, the presence of posterior synechia, narrow angle or shallow anterior chamber, currently on any miotic drugs, past history of any ocular injuries or intraocular surgeries including laser treatment, current infectious eye diseases, and being diagnosed with arterial hypertension, cardiac diseases or angle closure glaucoma. All participants were informed about the details of the study before signing the consent. Their eyes were inspected to detect the excluding conditions. The sample size was calculated by Query Advisor program. A total number of 40 patients were required to verify that the time spent for pupil dilatation was different between groups by more than 10 min.

Poor pupil dilatation is recognized in diabetic patients. The enrolled patients were therefore classified as diabetics or non-diabetics before being allocated to two groups by stratified block randomization. This measure distributed diabetic patients equally into each study group. Sequence of patient allocation was prepared by a computer program and sealed in envelopes beforehand. After the informed consent was signed, the envelope was opened and the patient was allocated accordingly.

The study drugs were prepared by two pharmacists. The first one set up a group of four drug bottles tagged with a green label numbering 1 to 4. Each bottle contained 0.75% tropicamide and 2.5% phenylephrine. This solution was prepared by mixing 3 mL of 1% tropicamide (Mydriacyl, Alcon Couvreur, Puurs, Belgium) with 1 ml of 10% phenylephrine (Phenylephrine, Biolab, Samutprakarn, Thailand). The resultant mixture was clear and colorless. The second pharmacist prepared another set of four drug bottles tagged with a red label numbering 1 to 4. The bottles numbered 1 and 3 contained 1% tropicamide. Bottles numbered 2 and 4 contained 10% phenylephrine. Both solutions were clear and colorless. The patients and investigators were unaware of the drugs contained in any of the bottles.

After patient allocation, the pupil diameter was vertically measured with a pupil gauge under bright light without magnification. This was performed by a separate investigator who was young, non-presbyopic and well-trained to do it consistently. The pupil gauge comprises a sequence of multiple half-circles progressing in diameter from 2 to 12 mm in 1-mm step. The eye was illuminated with a flashlight during measurement. The intensity of illumination was at least bright enough to allow clear observation of the pupil. This also tested the pupil sustaining dilatation against bright light as under indirect ophthalmoscopy. The pupil diameter was compared to the size of these half-circles on the pupil gauge. A pupil smaller than the smallest half-circle was counted as 1 mm in diameter. In the same manner, a pupil larger than 2 mm but smaller than 3 mm was considered 2 mm in diameter, and so on. Blood pressure and pulse rate were recorded before starting eye drop application.

Patients in the first group received eye drops from the green bottles. One drop from each bottle was applied in sequence according to the number marked on the bottle. The interval between drops was 10 min. Patients in the second group received eye drops from the red bottles in the same manner. A total of four applications were planned for each patient. Measurement of the vertical pupil size was repeated every 5 min after the initial eye drop application, for 40 min. In case of bilateral pupil dilatation, measurement of pupil size was done in both eyes; however, only the data from the eye whose pupil responded slower was included in the statistical analysis. Blood pressure and pulse rate were followed every 15 min. Application of drops was stopped once the pupil diameter reached 7 mm in size. This eliminated unnecessary exposure of patients to the study drug.

Kaplan-Meier survival analysis was used to compare the success of pupil dilatation to 7 mm within 40 min. The difference of the mean pupil size at each time point was tested with unpaired t-test. Blood pressure and pulse rate were also compared with t-test. The difference was judged to be statistically significant when the *P*-value was 0.05 or lower.

## Results

The total number of patients participating in this study was 40. Twenty-three patients (58%) were female. Ages ranged from 35 to 75 years with mean±SD of 57.3±10.9 years. Sixteen patients (40%) were diabetics. Twenty-two patients were randomized to receive the mixture drug and the other 18 patients to the alternate drug group. Baseline data of these groups is shown in Table 1; there was no significant difference between groups.

**Table 1:** Baseline data of patients in each group

	Mixture group	Alternate drug group	*P*-value
Number of patients	22	18	
Sex: male/female	11/11	6/12	0.526
Age: mean ± SD	55.8 ± 10.3	59.2 ± 11.8	0.343
No. of patients with diabetes (%)	9 (40.9)	7 (38.9)	1.0
Pupil size: mean ± SD (mm)	1.7 ± 0.5	1.8 ± 0.4	0.473

**Figure 1:**
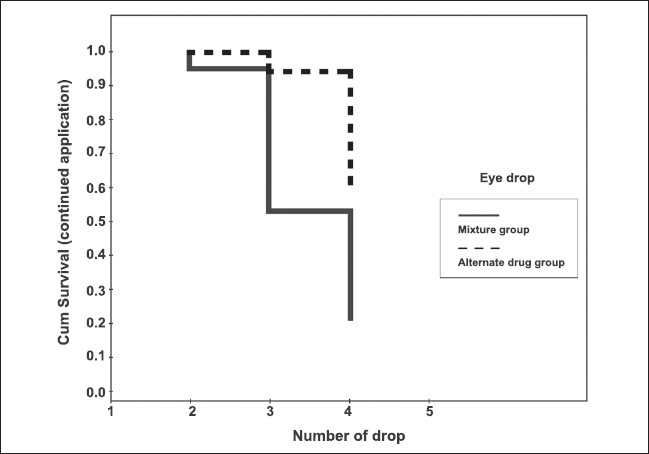
Kaplan-Meier survival curves demonstrate fractions of patients who still required drug application. The end of curves show fractions of patients whose pupil did not dilate to 7 mm

Within 40 min after initial eye drop application, the pupil could be successfully dilated to 7 mm in 17 patients (77%) of the mixture group compared to seven patients (39%) of the alternate drug group. One patient in the mixture group got such pupil dilatation with only two applications. An additional seven patients reached this goal by three applications. The remaining nine patients dilated to 7 mm after four applications. Only one patient in the alternate drug group got to this pupil diameter after three applications. This is summarized by Kaplan-Meier survival analysis as shown in Fig. 1. The proportion of patients requiring further applications, or unsuccessfully getting pupil dilatation to 7 mm was less in the mixture group. The difference was statistically significant by Log Rank test with *P*-value of 0.004.

**Figure 2:**
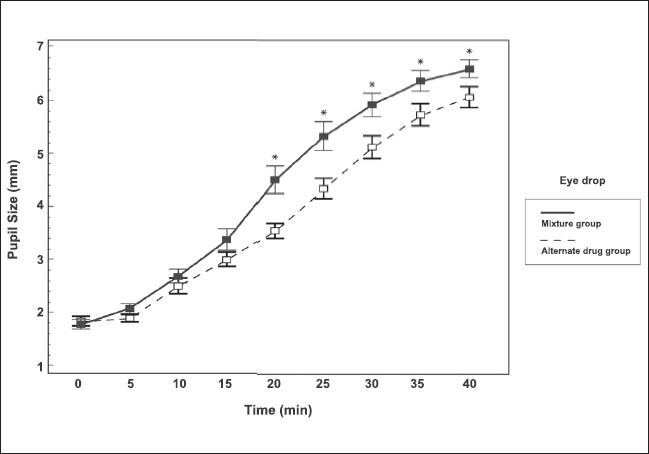
Progression of pupil diameter from baseline to 40 min after initial application. Vertical bars represent ±1 SEM. *indicate statistically significant difference of pupil size

The progression of pupil dilatation in both groups is shown in Fig. 2. The mean pupil size of the mixture group became significantly larger than in the standard group after 20 to 40 min. The *P*-values were 0.003, 0.007, 0.021, 0.036 and 0.050 at 20, 25, 30, 35 and 40 min respectively. The mean±SD of the pupil diameters at 40 min after drug application was 6.6±0.8 mm in the mixture group and 6.0±0.9 mm in the alternate drug group (*P* = 0.050). The pupil size was followed until 2 h after instillation. The mean±SD of the pupil size increased to 6.8±0.9 and 6.7±0.8 mm in the mixture and standard drug groups respectively. These were not significantly different.

Baseline blood pressure and pulse rate in both groups were similar. No significant change from baseline after drug application was found in either group. Blood pressure and pulse rate between the two groups was comparable at each time point. Details are shown in Table 2.

**Table 2:** Mean blood pressure and pulse rate at each time point

		Baseline	Minute 15	Minute 30	Minute 45
Systolic pressure (mm Hg)	Mixture group	129.8	125.4	124.7	126.9
	Alternate drug group	124.1	126.3	127.7	127.4
Diastolic pressure (mm Hg)	Mixture group	77.6	79.3	78.4	80.4
	Alternate drug group	79.1	79.8	79.5	80.9
Pulse rate (beats/minute)	Mixture group	80.2	80.9	80.8	79.3
	Alternate drug group	82.1	79.4	81.2	81.5

## Discussion

The quality of an intraocular examination depends on adequate pupil dilatation.[2] The ideal mydriatic agents should provide rapid dilatation of the pupil wide enough to permit thorough ocular evaluation, without having any significant local or systemic adverse effects.

The pupil is under the control of the autonomic nervous system. Parasympatholytic as well as sympathomimetic drugs have been used to dilate the pupil. The parasympathetic regulation dominates over the sympathetic effect in the control of the pupil.[3] Therefore, application of only the sympathomimetic drug is usually inadequate to sustain the pupil dilatation in bright light during indirect ophthalmoscopy. However, parasympatholytic agents alone may not provide sufficient pupil dilatation. Combination of both drugs offers greater pupil dilatation than single drug use.[4]

The mydriatric agents we commonly use are 1% tropicamide and 10% phenylephrine, administered alternately 10 min apart for two to three cycles. A study in non-diabetic patients showed that using these drugs could dilate the pupil from 4.4 to 7.6 mm at 60 min after initial application.[5] Repeated alternate application of drugs was practiced to accelerate pupil dilatation. Since phenylephrine may be associated with dangerous cardiovascular side-effects, using lower concentrations has been suggested in vulnerable patients. Although diluting the drug concentration reduces adverse effects, repeating alternate application poses confusion in sequencing when applying to many patients at the same time in a busy clinic. Combining drugs in a mixture simplifies this procedure.

In this study we used a mixture containing 0.75% tropicamide and 2.5% phenylephrine for pupil dilatation. It demonstrated superior efficacy over our standard application of 1% tropicamide alternating with 10% phenylephrine. The pupil was dilated quicker and wider with the mixture. In addition this offered convenience in clinical practice in that only one bottle of drug was required instead of two. This eliminated confusion in drug sequencing.

In case of multiple drugs application, the time interval between drops may affect the amount of drug absorption into the eye. The first drug applied requires some contact time for ocular penetration. The early application of the second drug may wash out the first one, reducing the chance to penetrate into the eye. Concerning the turnover rate of tear fluid, approximately 16% per minute, 10 min should elapse before any subsequent drop. This was the interval spacing between drops in this study. On the contrary when the identical drug is repeated, earlier or later, no washout effect will occur. This is another benefit of using a mixture.

Comparing these two regimens in this study, repeated application of the mixture administered both mydriatric agents each time whereas alternate application offered single drug at a time. In spite of lower drug concentrations in the mixture, the pupil response was more rapid and of greater magnitude. This suggested that sustaining the drug in the tear film resulted in greater ocular penetration than the bolus drug application.

The degree of pupil dilatation was shown to be significantly different between study groups starting 20 min after initial drug application. The ratios of the amount of tropicamide received in the mixture group to the standard drug group after the second, third and fourth application were 1.5:1, 2.25:2 and 3:2 respectively. The ratios of phenylephrine were 5:10, 7.5:10 and 10:20 at the same points of time. Therefore, the mixture group obtained more of the parasympatholytic agent but less sympathomimetic drug. Thus the dominant parasympathetic control of the pupil was more inhibited in the mixture group. Even with less amount of sympathomimetic agent, the mixture group obtained this drug 10 min earlier than the standard drug group. This resulted in faster pupil dilatation with the mixture.

There are several reports studying the effects of various mixtures of tropicamide and phenylephrine.[6–9] All these studies aimed to find the optimal low concentrations of both drugs in the mixture that provided adequate pupil dilatation. The concentrations varied from 0.125 to 1.0% for tropicamide, and 1.25 to 5.0% for phenylephrine. Apt *et al*. studied a combination of 0.5% tropicamide and 2.5% phenylephrine in patients aged between 16 to 84 years.[6] This mixture produced pupil dilatation to 7 mm within 60 min. Its competency was equal to a combination of 1% tropicamide and 2.5% phenylephrine. They also found that pupils of younger patients dilated better than those of the older. Krumholz *et al.* showed equivalent effects of solutions containing 0.5% tropicamide with either 2.5% or 1.25% phenylephrine.[9] All pupils reached at least 7 mm in diameter within 30 min. However, this study was conducted in young subjects 21 to 40 (median 23) years of age. Mean baseline pupil size was 3.0 mm. On the contrary, the participants in our study were older and included diabetics, as they were the majority of patients in our clinic who required pupil dilatation for ophthalmoscopy. This group of patients traditionally has small pupils and responds poorly to mydriatic drugs. This was the reason why we chose the concentrations of drugs we did in this study.

Measurement of the pupil size in our study was conducted under bright light. The purpose was to verify the sustainability of pupil dilatation under indirect ophthalmoscopy. The way we used the pupil gauge to determine the pupil size as described was also attributed to the smaller baseline pupil diameter when compared to other studies. The pupil gauge is not as delicate as a pupillometer to distinguish subtle differences in pupil size. The measurement was performed external to the cornea. So it was not the actual measurement of the pupil. The magnification factor had to be used to convert this measurement to the actual pupil size. Besides, the scale grade of 1 mm in the pupil gauge was crude. These were the limitations of this study. However, the method we used, that is the pupil gauge, was reproducible and sufficient to detect clinically meaningful data for comparison between two regimens.

In conclusion, the mixture containing 0.75% tropicamide and 2.5% phenylephrine was effective in pupil dilatation. It provided rapid and successful pupil dilatation for ophthalmoscopy, more than the alternate application of 1% tropicamide and 10% phenylephrine. Besides, application of this mixture made the process of pupil dilatation easier and quicker, shortening the time spent in the outpatient eye service. The questions waiting for answers should be whether a single dose could offer equivalent outcome and whether prior application of anesthetic eye drop could enhance its effect.
